# A Case of Refractory Mycoplasma Pneumonia With Extra-pulmonary Manifestations in a Community-Acquired Setting

**DOI:** 10.7759/cureus.95193

**Published:** 2025-10-22

**Authors:** Shweta S Acharya, Burabattula B Shashank, Ashok Kumar, Pankhuri Kumari, Ashok Kumar

**Affiliations:** 1 Internal Medicine, Max Smart Super Specialty Hospital, New Delhi, IND; 2 Internal Medicine, Max Smart Super Specialty Hospital, New Delhi, IND; 3 Microbiology, Amrita Hospital Faridabad, Faridabad, IND; 4 Internal Medicine, Max Smart Super Speciality Hospital, New Delhi, IND

**Keywords:** community aquired pneumonia, extra-pulmonary manifestation, hemolytic anaemia, macrolide-resistant mycoplasma pneumoniae, mycoplasma pneumonia, refractory mycoplasma pneumonia

## Abstract

This case highlights a young female in her early twenties who presented with fever and persistent dry cough of one week duration. After ruling out other conditions she was subsequently diagnosed as Mycoplasma pneumonia. It is one of the important causes of atypical pneumonia. The manifestations can range from mild upper respiratory tract infections to severe pulmonary and extra-pulmonary manifestations. The patient's clinical course was complicated by systemic immune activation, which manifested as elevated inflammatory markers, radiological and clinical worsening and development of autoimmune hemolytic anemia. Prompt identification and management with appropriate antimicrobials and other supportive therapy along with timely use of steroid led to steady clinical improvement. This case underlines the importance of considering atypical pathogens in febrile respiratory illnesses and the vigilance for early recognition of systemic immune phase complications.

## Introduction

Mycoplasma pneumoniae is one of the causes of community-acquired pneumonia and comes under atypical pneumonias, particularly affecting adolescents and young adults under the age of 40 years. Epidemics occur every three to seven years, especially in crowded areas [1]. It accounts for up to 35% of cases of community-acquired pneumonia and is transmitted via respiratory droplets, with outbreaks often reported in community settings such as schools, dormitories, and military facilities. The clinical presentation can vary widely, ranging from mild upper respiratory tract symptoms to more severe lower respiratory involvement, and is often insidious in onset with fever and persistent dry cough. Due to its often subtle presentation and overlapping features with viral respiratory illnesses, diagnosis can be delayed without a high index of suspicion. The incubation period is approximately two to three weeks. Although often self-limiting, Mycoplasma pneumoniae can cause extrapulmonary complications such as hemolytic anemia, encephalitis, skin rashes, and many others [2]. Diagnosis is made with strong clinical suspicion and confirmed by serology and polymerase chain reaction (PCR) methods. Important differential diagnoses include viral pneumonia, Chlamydia pneumonia, Legionella pneumonia, Coxiella pneumonia, bacterial pneumonias, and tuberculosis. This case report discusses the clinical presentation of Mycoplasma pneumoniae in a previously healthy young female in her early twenties who was later diagnosed with refractory Mycoplasma pneumonia.

## Case presentation

A female patient in her early twenties presented with complaints of fever for the past week, which was high-grade, continuous, and up to 104 degrees Fahrenheit. The fever responded only transiently to the paracetamol tablet. She also had a cough from the past week, which developed along with the fever, which was dry in nature with no diurnal or seasonal variation. The cough increased from three days before admission, associated with throat pain. She also developed two episodes of loose stools one day before admission, which were watery in consistency and did not contain blood or mucous. Loose stools were associated with crampy abdominal pain after passing stools. She has been having other symptoms like headache, bodyache, and reduced appetite. There was no significant past medical history. The travel history included a recreational trip with office colleagues, where a few of them were also having high-grade fever. On examination at admission, she was having a pulse of 87/ min, BP of 110/70 mmHg, SpO2 of 99% at room air, and bilateral crepitations on auscultation, which was heard on the infrascapular, infraaxillary, and infra-mammary areas. Reports showed complete blood count (CBC), liver function test (LFT), kidney function test (KFT) were within normal limits, and raised erythrocyte sedimentation rate (ESR) and C-Reactive protein (CRP). The typhoid IgM test for typhoid was negative (Table 1).

**Table 1:** Investigations done throughout the hospital stay. IgM- immunoglobulin M, IgG- immunoglobulin G, HCV IgG- hepatitis C virus immunoglobulin G, HBsAg- hepatitis B virus surface antigen, HIV- human immunodeficiency virus, NT ProBNP- N terminal pro brain natriuretic peptide, AFB- acid-fast bacilli, CMV-cytomegalovius, PCR/ RT-PCR- polymerase chain reaction/ reverse transcription-PCR, CT - computed tomography, CTPA with DVT protocol-  computed tomography pulmonary angiography with deep vein thrombosis protocol.

Investigations	Patient’s value	Normal range with units
Hemoglobin (Hb) on 7 th day of illness	10.7 g/dL	13-17 g/dL
Total Leucocyte Count (TLC) on 7 th day of illness	5.1x 10^9^/L	4.0-10.0 x 10^9^/L
Platelet Count (on 7 th day of illness)	260 x 10^9^/L	150-400 x 10^9^/L
Erythrocyte sedimentation rate (ESR)	43 mm/hour	0-20 mm/ hour
C-reactive protein (CRP)	354.69 mg/L	0-5 mg/L
Typhidot IgM	Negative	
Malaria antigen	Negative	
H1N1 PCR	Infuenza A, B, RSV (Respiratory Syncytial Virus), H1N1- Negative	
Prothrombin Time (PT)	15.2 sec	10.2-13.6 sec
International Normalized Ratio (INR)	1.3	
Total bilirubin (on day of admission)	0.66 mg/dL	0.3-1.2 mg/dL
Indirect bilirubin (on day of admission)	0.48 mg/dL	0.1-1.0 mg/dL
Aspartate aminotransferase (AST)	36 IU/L	15-41 IU/L
Alanine aminotransferase (ALT)	24 IU/L	17-63 IU/L
Albumin	3.3 g/dL	3.5-5.0 g/dL
aPTT (activated partial thromboplastin time)	36.3 sec	24.7-34.8 sec
Serum Creatinine	0.6 mg/dL	0.5-1.04 mg/dL
Serum Sodium	133 mmol/L	136-144 mmol/L
Serum Potassium	3.63 mmol/L	3.6-5.1 mmol/L
Serum calcium	7.6 mg/dL	8.8-10.6 mg/dL
Hemoglobin (Hb) on 13th day of illness	7.8 g/dL	13-17 g/dL
Total Leucocyte Count (TLC) on 14th day of illness	7.9x10^9^/L	4.0-10.0 x 10^9^/L
Platelet Count (on 14th day of illness)	451x 10^9^/L	150-400 x 10^9^/L
Mycoplasma IgM	> 27 AU/mL ( positive)	<10.0 AU/mL
Mycoplasma IgG	3.95 AU/mL	<10 AU/mL
Total Bilirubin (on day 14 th of Illness)	2.16 mg/dL	0.3-1.2 mg/dL
Indirect Bilirubin (on day 14 th of Illness)	1.57 mg/dL	0.1-1.0 mg/dL
Serum Lactate Dehydrogenase (LDH) on day 14 th of Illness	453 IU/L	0-247 IU/L
Aspartate aminotransferase (AST)- (on day 14 th of Illness)	53 IU/L	15-41 IU/L
Alanine aminotransferase (ALT)- (on day 14 th of Illness)	208 IU/L	17-63 IU/L
Direct coomb’s test (column agglutination)	Positive (3+)	Negative
Cold agglutinin Test, Serum	Positive	Negative
HCV IgG	0.02 S/CO	<0.9 S/CO
HBsAg	0.10 S/CO	<0.9 S/CO
HIV I &II, Serum	Non-reactive (<1.0 S/CO)	<1.0 S/CO
Anti-nuclear antibody (ANA)- immunofluorescence	Negative (1:80)	
Troponin I	0.004 ng/mL. (<0.02 ng/mL)	
Creatinine Kinase MB (CK-MB)	1.1 ng/mL. (0.6-6.3 ng/mL)	
NT- ProBNP	27.9 pg/mL. (<95.3 pg/mL)	
D-dimer (Quantitative)	3068 ng/mL. ( 0-248 ng/mL)	
Bronchoalveolar lavage (BAL)	BAL Gram Stain : No Organism Seen BAL Fungus Examination: No fungal elements seen. BAL Fungus Culture: No growth BAL GeneXpert MTB/RIF Ultra: Negative BAL for Malignant Cells: Atypical squamoid cells present. BAL AFB stain: No AFB seen on ZN (Ziehl-Neelsen) smear. BAL Culture & Sensitivity: No growth. BAL Galactomannan : 0.10 ug/L. ( <0.5 ug/L) BAL Biofire (lower respiratory pannel, multiplex PCR): Mycoplasma detected. BAL CMV DNA : <140 copies/mL ( negative value <140 copies/mL) BAL pneumocystis jiroveci RT PCR: Not Detected	
Transbronchial needle aspiration (TBNA)	TBNA GeneXpert MTB/RIF Ultra: Negative TBNA AFB stain: No AFB seen on ZN smear. TBNA Culture & Sensitivity: No growth	
Endobronchial Ultrasound (EBUS)	Mediastinal node (A,B) Features are suggestive of reactive node.	
Urine routine and microscopy	Normal	
Urine culture	No growth	
Paired blood culture	No growth	
Chest X-ray on day 7 of illness	Confluent areas of consolidation are seen in bilateral lower lung zones.	
CT thorax	bilateral large confluent areas of dense consolidation with air bronchogram predominantly involving lower lobes, few patchy dependant areas in right upper and middle lobes. Associated bilateral mild pleural effusion, more on right side. Mildly enlarged discrete non-calcified mediastinal lymph nodes. Likely infective aetiology.	
CTPA with DVT protocol	No evidence of pulmonary artery thrombosis or deep vein thrombosis in bilateral lower limbs is appreciated.	

**Figure 1:**
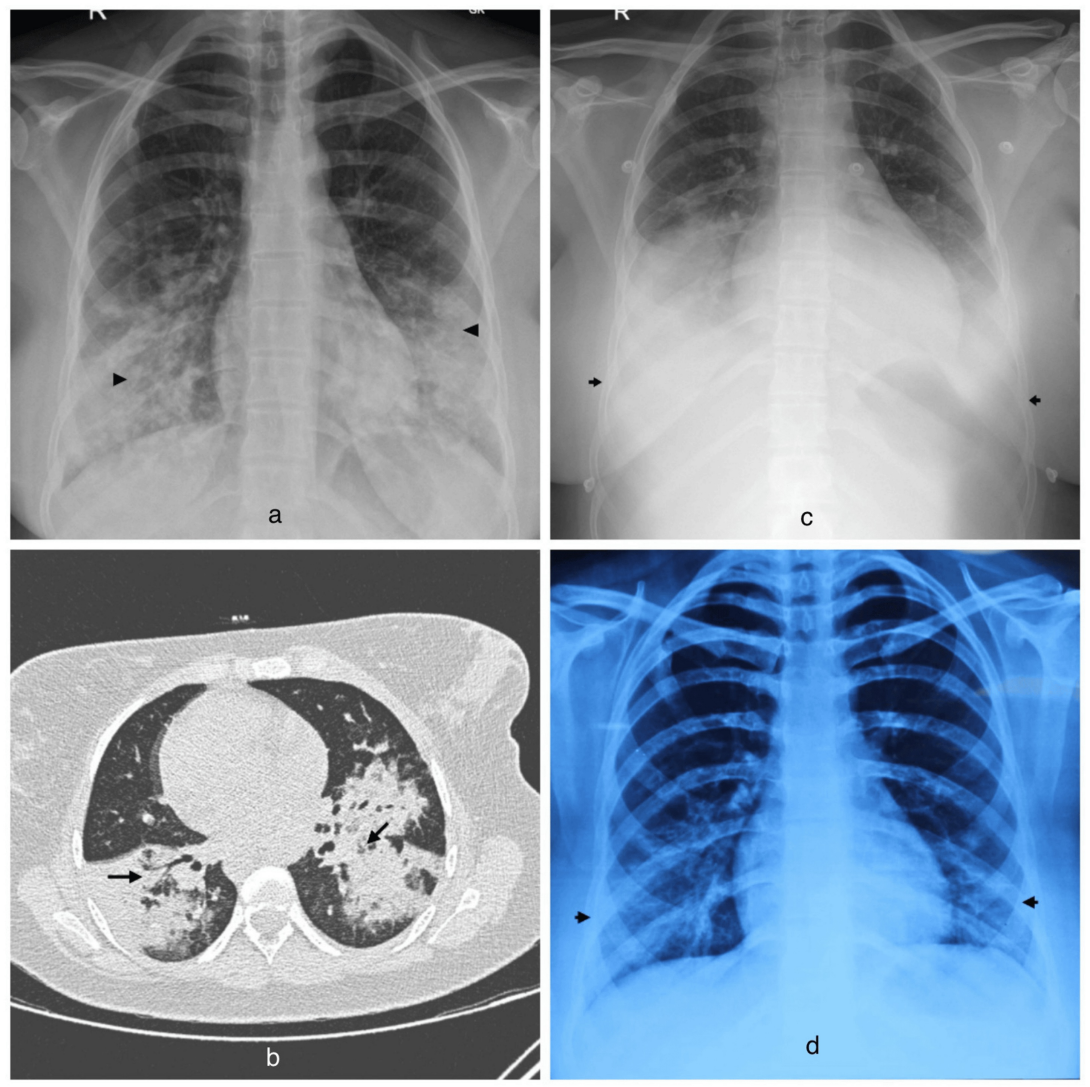
a. Chest X-ray on the day of admission (7th day of illness), b. High-resolution computed tomography of the chest, c. Chest X-ray on the 14th day of illness, d. Chest X-ray obtained two weeks after discharge a. Chest X-ray on the day of admission, which is the 7th day of illness, shows confluent areas of consolidation in the bilateral lower lung zones (black arrowheads). b. High-resolution computed tomography of the chest reveals bilateral large confluent areas of dense consolidation with air bronchograms, mainly involving the lower lobes (black arrows), along with a few patchy dependent areas in the right upper lobe. There is an associated bilateral mild pleural effusion, more pronounced on the right side, and mildly enlarged, discrete, non-calcified mediastinal lymph nodes. The findings suggest a likely infective etiology. c. Chest X-ray on the 14th day of illness demonstrates bilateral lower zone confluent opacities and consolidation, with an obscured costophrenic angle (black arrows). d. Chest X-ray obtained two weeks after discharge shows resolution of bilateral lower zone opacities (black arrows) and a clear, bilaterally normal costophrenic angle.

The chest X-ray showed bilateral lower zones heterogeneous opacities (Figure 1a). She was started on treatment lines of community-acquired pneumonia and was given Inj piperacillin tazobactam and Tab azithromycin, along with other supportive treatment including nebulization, mucolytics, intravenous fluid, and Inj paracetamol. Her urine and paired blood cultures were sterile. With the above treatment, the frequency and intensity of fever, as well as dry cough, did not show any significant improvement even on the third day of starting the antibiotics. Thus, high-resolution computed tomography of the chest (HRCT) was done, which showed bilateral large confluent areas of dense consolidation with air bronchogram predominantly involving the lower lobes, and a few patchy dependent areas in the right upper and middle lobes. Mildly enlarged discrete non-calcified mediastinal lymph nodes (Figure 1b). H1N1 polymerase chain reaction (nasal swab) was negative. Pulmonology consultation was taken, and Bronchoscopy with Bronchoalveolar Lavage (BAL) was done, which showed BAL biofire multiplex polymerase chain reaction-positive for Mycoplasma. Mycoplasma IgM antibody also came positive. Respiratory secretions for Cytomegalovirus (CMV), Pneumocystis jirovecii (PCP), and Mucormycosis, reverse transcriptase polymerase chain reaction (RT-PCR), as well as BAL culture sensitivity were negative. In view of persisting fever and dry cough, tablet azithromycin was stopped, and the patient was started on capsule doxycycline on the fourth day of admission. On the seventh day of hospitalization, which is the 14th day from the onset of fever, the patient developed a fall in SpO2 (oxygen saturation) with an increase in breathlessness and was started on oxygen supplementation. Repeat X-ray chest showed bilateral significant pleural effusion (Figure 1c). Her creatine kinase myocardial isoenzyme (CK-MB), troponin-I, electrocardiogram (ECG), as well as 2D echocardiography were within normal limits. Her CBC also showed a fall in hemoglobin with features suggestive of hemolysis - direct Coombs’ test and cold agglutinin were positive. Her lactate dehydrogenase enzyme (LDH) and indirect bilirubin were also raised. Anti-nuclear antibodies (ANA) by immunofluorescence were negative. Her liver enzymes also showed a rise in aspartate aminotransferase (AST) and alanine aminotransferase (ALT). Her C-reactive protein (CRP) and D-dimer were raised. Computed tomography pulmonary angiography (CTPA) with deep vein thrombosis (DVT) protocol was done to rule out pulmonary embolism as a cause of worsening breathlessness and desaturation, which showed no evidence of pulmonary artery thrombosis or deep vein thrombosis in the bilateral lower limbs. She was started on intravenous methylprednisolone 1mg/kg two times a day intravenously for three days, followed by gradual tapering. Gradually, her effort tolerance increased, and oxygen supplementation was removed. She could maintain adequate oxygen saturation at room air. Hemoglobin improved, and LDH reduced. She was then discharged subsequently on oral methylprednisolone. On outpatient department (OPD) follow-up after two weeks of discharge, the patient was asymptomatic, and her repeat chest x-ray showed significant resolution in the opacities (Figure 1d).

## Discussion

Mycoplasma pneumoniae is one of the commonest causes of respiratory tract infections affecting humans, commonly upper respiratory tract infections and, in some cases, as pneumonia. It is one of the causes of atypical pneumonia. However, in some patients, it may have extrapulmonary manifestations [3]. Mycoplasma measures 100 μm in diameter, and it lacks a cell wall, which makes the organism undetectable on Gram staining as well as resistant to beta-lactam antibiotics. Mycoplasma is a fastidious organism and needs special culture media for isolation. Moreover, its isolation from respiratory secretions may not indicate acute infection at that point in time, as mycoplasma may be excreted from the respiratory tract for a few weeks after the acute infection [4]. The majority of patients present with minor respiratory illness, including pharyngitis and tracheobronchitis, which is usually self-resolving. Around 3%‐13% of infected individuals develop pneumonia. Infection spreads via respiratory droplets and has an incubation period of two to four weeks. At-risk age groups are mostly between five to 20 years [5].

Mycoplasma pneumonia

The patient may present with symptoms of fever, malaise, headache, and cough. Some patients may also have a sore throat, nasal symptoms, and chest discomfort. Patient characteristically presents with a persistent cough, which might increase in frequency and intensity over 1‐2 days. On examination patient might have crepitations on chest auscultation, cervical lymphadenopathy, and may also have pharyngeal erythema [6]. On chest x-ray, consolidation in mid to lower zones is seen, followed by ground glass opacities. Macrolide-resistant Mycoplasma pneumonia (MR‐Mp) is defined as fever persisting for more than 72 hours without improvement in clinical and radiologic findings after macrolide antibiotics [7]. Another entity is Refractory Mycoplasma pneumoniae pneumonia, which is defined as fever persisting for more than seven days despite appropriate antibiotics and or worsening of clinical or radiological findings. Such patients have an increased incidence of extra-pulmonary complications, which is supposed to be due to exaggerated host immune response rather than direct mycoplasmal damage. Lactate dehydrogenase (LDH) has been considered as a biomarker of refractory Mycoplasma pneumoniae, which gets upregulated [8].

Extra-pulmonary manifestations

Mycoplasma infections cause various extrapulmonary complications, such as neurological, cardiovascular, dermatological, gastrointestinal, and hematological. Neurologic complications occur in less than 1 per 1000 cases and include encephalitis, meningoencephalitis, Guillain‐Barré syndrome, etc. CNS involvement occurs due to direct invasion of organisms or generation of toxins or due to production of autoantibodies directed against the neurons and myelin [9]. Cardiovascular manifestations include pericarditis, endocarditis, and myocarditis. Dermatological manifestations include Stevens‐Johnson syndrome, erythema nodosum, and erythema multiforme. Liver dysfunction may occur around seven to 10 days after the onset of fever, which is reflected by elevation of aspartate aminotransferase and, less frequently, by elevation of alanine aminotransferase. Hematological manifestations include autoimmune hemolytic anemia, which is due to cross-reactivity of antibodies to M. pneumoniae antigens to red blood cells. Other manifestations include hemophagocytic syndrome, cytokine‐storm disorders, and disseminated intravascular coagulation. In some patients, mucositis, otitis media, and glomerulonephritis due to immune complex deposition in the glomeruli are also reported in some cases [10].

Workup

Definitive diagnosis of Mycoplasma is by culture, but it is rarely done as it needs special culture medium and takes seven to 21 days to provide results. Serological methods include Mycoplasma antibodies, IgM, and IgG. Other methods, such as particle agglutination (PA), complement fixation (CF), are also available. The loop-­ mediated isothermal amplification (LAMP) assay is a nucleic acid amplification method for the rapid detection of Mycoplasma by throat swab or sputum is also available [11]. Another rapid antigen kit is available for Mycoplasma ribosomal protein L7/L 12 detection (ribotest mycoplasma). It is based on an immunochromatographic assay and has a sensitivity of 60% as compared to PCR [12]. Hemolysis workup includes positive Coomb test, raised reticulocyte count, and raised Cold agglutinin titers in more than 50% of cases with Mycoplasma pneumonia. Total leucocyte count is normal in 75% to 90% of cases. Chest x-ray findings include unilateral or bilateral patchy consolidation or reticulonodular pattern [13]. High-resolution computed tomography of the chest may show centrilobular nodular and tree-in-bud pattern, peri-bronchial thickening, patchy distribution, ground glass opacities, lobular opacity, and pleural effusion in 20% of cases [14].

Treatment recommendations

As Mycoplasma pneumoniae lacks a cell wall, antibiotic groups effective against the organism include macrolides, doxycycline, and fluoroquinolones [15]. Macrolides are the first drug and have 50 % bioavailability to oral dose and can achieve 300 times tissue concentration in the epithelial lining fluid, alveolar macrophages, and neutrophils [16]. Oral clarithromycin for 10 days or oral azithromycin for five days is preferred [17]. Fluoroquinolones such as levofloxacin have also been used for 7-14 days [18]. Macrolide-resistant Mycoplasma pneumonia should be treated with tetracyclines (doxycycline or minocycline ) for 10 days and fluoroquinolones for seven to 14 days [19]. Treatment of refractory Mycoplasma pneumonia includes systemic corticosteroids such as methylprednisolone in the dose of 2mg/kg/ day in divided doses for 3 days has shown good recovery. In case of fever persisting for over 72 hours or features of clinical deterioration, an increase in the dose of methyl prednisolone to 4- 6 mg/kg/ day in divided doses or IVIG in the dose of 400mg/kg/day for three days is considered [20].

## Conclusions

Mycoplasma pneumonia can have a wide spectrum of presentation, ranging from mild upper respiratory tract infection to severe refractory mycoplasma pneumonia with extrapulmonary manifestations. In cases of acute respiratory febrile illness that is not responding to first-line treatment or showing clinical deterioration, Mycoplasma pneumonia should be considered as an important cause, and we have to be vigilant about systemic immune activation syndrome. Prompt diagnosis and timely treatment can shorten hospital stay and reduce morbidity and mortality.
